# The Effect of Enoxaparin and Clopidogrel on Survival of Random Skin Flap in Rat Animal Model

**Published:** 2012-07

**Authors:** Mohammad Javad Fatemi, Kamal S Forootan, Seyed Ziaaddin S Jalali, Seyed Jaber Mousavi, Mir Sepehr Pedram

**Affiliations:** 1 Associate Professor of Department of Plastic Surgery, Burn Research Center, Hazrate Fatemeh Hospital, Tehran University of Medical Sciences, Tehran, Iran.; 2 Plastic Surgeon, Vali Asr Police Hospital, Tehran, Iran.; 3 Department of Community Medicine, Burn Research Center, Tehran University of Medical Sciences, Tehran, Iran.; 4 Veterinary Surgeon, School of Veterinary Medicine, Tehran University, Tehran, Iran.

**Keywords:** Enoxaparin, Clopidogrel, Survival, Skin flap, Rat

## Abstract

**BACKGROUND:**

Necrosis of skin flaps is considered as an important complication in reconstructive surgery. We conducted an experimental study to investigate the efficacy of low-molecular weight heparin, clopidogrel and their combination to improve the flap survival.

**METHODS:**

Forty male, adult Sprague-Dawlay rats were divided randomly into 4 groups. Standard rectangular, distally based dorsal random pattern skin flap was elevated. To prevent the graft effect, a sterile sheet was put under the flap. No pharmacological agent was administered for the control group. In group 2, single subcutaneous dose of enoxaparin (3.2 mg/kg) was immediately administrated after surgery. In group 3, clopidogrel (25 mg/kg) was given orally for 7 days. In group 4, both enoxaparin and clopidogrel were administrated. The rats were evaluated on post-operative day 7 for viable and necrotic portions of flaps.

**RESULTS:**

The mean and SD of necrosis was 17.79+2.5 cm in the control group, 16.20±3.1 cm in low-molecular weight heparin, 15.25+3.8 cm in combined therapy group and 13.69+2.7 cm in clopidogrel group. Clopidogrel was the only pharmaceutical agent that produced a significant increase in the flap survival area.

**CONCLUSION:**

Clopidogrel may be an effective pharmaceutical agent that significantly increases viability of random skin flaps in rats, but low-molecular weight heparin and their combination did not have any significant beneficial effects.

## INTRODUCTION

Random pattern skin flaps are common in various fields of plastic and reconstructive surgery and necrosis is its important possible complication. Patients experiencing flap necrosis need a prolonged wound care. Also, these patients frequently need multiple operations and experience severe scar formation. These outcomes are associated with more hospitalization, longer work-off periods, unsatisfied results and increased medical costs.^1^,^2^

The main causes of flap necrosis can usually be attributed to specific problems in the hemodynamic of the flap including arterial flow insufficiency, venous congestion, or possibly a combination of the two mechanisms. To solve these problems and improve the survival of skin flaps, numerous surgical methods and pharmaceutical agents have been proposed and thoroughly studied.^3^

Pharmacological modulation of flap survival includes sympatholytics, vasodilators, hemorhological agents, prostaglandins, anticoagulants, glucocorticoids and free-radical scavengers. These agents are suggested to be beneficial for flap circulation and survival. Most of these agents produce vasodilation and improve the circulation of the flap. For this purpose, different pharmacological agents such as dipyrone, intravenous and topical nitroglycerin, pentoxifylline, minoxidil allopurinol, desferrioxamine, streptokinase, aspirin, urokinase, tissue plasminogen activator (tPA), acylated plasminogen-streptokinase activator complex (APSAC), dimethyl sulfoxide (DMSO), diltiazem, verapamil, dextran, dipyridamole, histamine, hydralazine, heparin, H-dopa, goanethidine, reserpine, propranolol, and prostaglandins have been used by different methods. Some drugs are used topically, others by intra-flap injection, or systemically.^1^-^4^

Other methods such as mechanical preconditioning and nerve stimulation, electrical stimulation, ultrasound, hyperbaric oxygen, laser and different temperature levels have also been used to improve flap survival.^2^,^4^,^5^ The only method of proven efficacy in improving survival of skin flap is surgical delay Procedure.^6^

The effect of anticoagulants in enhancing tissue blood supply is beyond any doubt. Heparin, aspirin and clopidogrel are the most used medicines.^7^ Low molecular weight heparins (LMWHs) were used in treatment of ischemic heart disease, myocardial infarction and prophylaxis of thromboembolism in different fields of surgery.^7^ Low-molecular-weight-heparin (LMWH) is a new class of synthetic anticoagulant that selectively binds and potentiates antithrombin III, thereby specifically inhibiting factor Xa in the coagulation cascade. This is opposed to other anticoagulants (ie, heparin), which binds to multiple factors in the coagulation cascade.^7^

Heparin has been used with variable success rate in the literature for the salvage of ischemic pedicle flaps. In a rabbit model, intravenous heparin that was administered in varying dose could significantly improve flap circulation and survival even if administered 6 hours after flap dissection.^8^ In a pig model using axial random flaps, a constant infusion of heparin could not produce any improvement in flap survival.^9^

In Shalom *et al. *study, heparin did not have a beneficial effect on flap survival.^10^ Miyawaki *et al. *showed that in animal models, subcutaneous doses of LMWHs improved flap survival.^11^ In Bielecki *et al. *study, prophylactic dose of this drug did not have any beneficial effect in flap survival.^4^ In Torkvistl *et al. *study, treatment with heparin in the clinical dose- range markedly increased the viability of skin flaps in the rat.^12^ In Celik *et al. *study, local heparin produced significant improvement of flap survival.^2^ In Chung *et al. *study, both fondaparinux and enoxaparin had beneficial effects on congested skin flaps.^13^

Clopidogrel is a thienopyridine derivative that inhibits platelet aggregation and is a prodrug that needs a hepatic cytochrome P450 enzyme termed P2C19 to change into its bioactive metabolites and inhibits platelet aggregation through irreversible blockade of P2Y12 (ADP) receptors on platelets. Much of the clopidogrel dose undergoes esterase deactivation, and therefore only a small portion is metabolized to its active moiety in the liver. Peak plasma metabolite concentration occur after 1 hour and bioavailability is unaffected by food.^14^,^15^

Clopidogrel has been shown to markedly reduce the incidence of both arterial and venous thrombi in animal models. It is a key antiplatelet therapy in patients with acute coronary syndrome or undergoing percutaneous coronary intervention. Also it has beneficial effects in peripheral arterial and cerebrovascular diseases.^14^,^16^

There are lots of clinical and experimental studies with different results about heparin effect on flap survival, but there are only two randomized controlled animal studies about the effect of clopidogrel on flap survival and proved beneficial effects of this agent.^17^,^18^ Due to this controversy about heparin and also few articles about clopidogrel, this study was conducted to investigate the efficacy of low- molecular weight heparin, clopidogrel, and their combination on improvement of flap survival.

## MATERIALS AND METHODS

The study is a randomized controlled double blind animal study. Forty male, adult Sprague- Dawley rats each weighing between 350 to 400 g were enrolled. Rats were randomly assigned to reactive either clopidogrel, low molecular weight heparin and combination of them (each group contained 10 rats). A control group of 10 rats was also included in the study. The Health guidelines of Tehran University of Medical Sciences for the care and use of laboratory an- imals were followed throughout the study. All animals received food and water in an unrestrained manner.

The surgeries were performed by one surgeon. Rats were anesthetized intra-muscularly with ketamine (90 mg/kg; Alfasan, Netherlands) and xylazine (9 mg/kg; Alfasan, Netherlands). Following the induction of general anesthesia, the dorsal regions were shaved and then prepared with providing iodine solution. Each animal was placed in prone position and a standard template was used to mark the design of a 3×11 cm rectangular, distally based dorsal flap. Then full thickness, random pattern skin flap including panniculus carnousus was elevated. To prevent the graft effect, a sterile sheet (incifilm, pharmaplast, Alexandria, Egypt) was put under the flap and reapproximation was performed with interrupted 4-0 nylon (Supa, Iran) sutures.

No pharmacological agent was given to the control group (group 1). In group 2, low molecular weight heparin (Clexan, Sanofi- Aventis, France) was administrated (3.2 mg/kg) as a single subcutaneous dose immediately after surgery. In groups 3, clopidogrel (Plavix, Sanofi-Aventis, France) was given orally (25 mg/kg) by gavages dissolved in 1 ml normal saline daily for 7 days, the first dose 6 hours before flap elevation. In group 4, both low molecular weight heparin and clopidogrel were administrated using the same techniques in groups 2 and 3.

The flaps were monitored daily for hematoma formation, dehiscence or cellulites. The rats were evaluated on post-operative day 7 for viability of flaps. They were placed in prone position and digital photography (Nikon L300, Macro lens 60 mm magnification 1:10, 80 cm distance) was taken with a ruler in the field. Measurements of viable flaps were performed with Image J software (NIH, USA). The rats were then sacrificed by overdoses of phenobarbital. The mean area of flap survival was calculated for each group and compared. All statistical analysis was performed using a t- test, with a significance level set at *P*<0.05.

## RESULTS

**Fig. 1:**
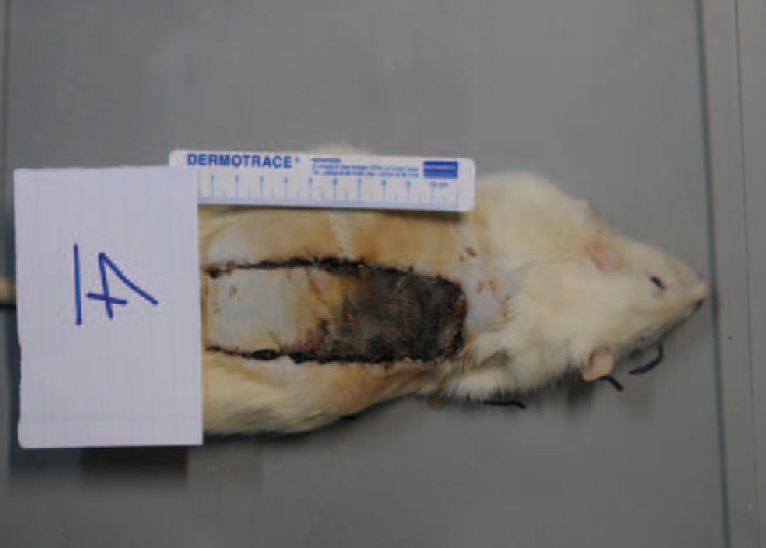
Flap elevation and putting an incifilm under it

One rat from the control group, one from the clopidogrel group, and 3 rats from the clopidogrel- heparin group were expired during study. In the remaining animals, none of the flaps demonstrated evidences of cellulitis, wound dehiscence or hematoma. The regions of survival and necrosis were clearly demarcated in every flap on day 7 (Figure 1). The results of the mean areas of flap survival and necrosis are summarized in Table 1 and a comparison of the mean percentage of the necrotic are is shown in Table 2. The animals in the control group (N=9) had a mean flap necrosis area of 17.76±2.5 cm2 and in the clopidogrel group (No=9) had a mean flap necrosis area of 13.69±2.7 cm. The animals in the low- molecular weight heparin group (N=10) had a mean flap necrosis area of 16.2±3.1 cm2. Finally, The animals in the combined clopidogrel low molecular weight heparin group (N=7) had a mean flap necrosis area of 15.25±3.8 cm2. The mean survival percentage of flaps

$\left(\frac{\mathrm{survival area}}{\mathrm{total area}}\times 100\right)$

were 46% in the control group, 52% in the low molecular weight heparin group, 60% in the clopidogrel group, and 56% in the combined therapy group. Clopidogrel was the only pharmaceutical agent that produced significant increase in the flap survival area and decreased the necrosis of the flap (*P*<0.05).

**Table 1:** The results of the mean areas of flap survival and necrosis

**Descriptive Statistics**						
**Group**		**N**	**Minimum**	**Maximum**	**Mean**	**Std. Deviation**
Control	Total surface of flap (cm2)	9	28.56	37.50	32.91	2.59
	Necrosis surface of flap (cm2)	9	12.69	22.88	17.77	3.16
	Ratio	9	0.38	0.73	0.54	0.09
	Valid N (Listwise)	9				
Clexan	Total surface of flap (cm2)	10	27.15	38.83	33.98	3.59
	Necrosis surface of flap (cm2)	10	12.85	23.40	16.20	3.13
	Ratio	10	0.36	0.65	0.48	0.09
	Valid N (Listwise)	10				
Clopidogrel Bisulfate (plavix)	Total surface of flap (cm2)	9	30.29	38.15	34.21	2.43
	Necrosis surface of flap (cm2)	9	9.29	18.32	13.70	2.79
	Ratio	9	0.28	0.48	0.40	0.07
	Valid N (Listwise)	9				
Clexn and Clopidogrel Bisulfate (plavix	Total surface of flap (cm2)	7	32.59	34.12	33.08	0.52
	Necrosis surface of flap (cm2)	7	11.57	21.26	15.25	3.85
	Ratio	7	0.35	0.62	0.46	0.11
	Valid N (Listwise)	7				

**Table 2:** Comparison of the mean percentage of the necrotic are

**ANOVA test**						
		**Sum of** **Squares**	**D/F**	**Mean** **Square**	**F test**	**Significance**
Total surface of flap (cm2)	Between Groups	10.94	3	3.65	0.51	0.67
	Within Groups	218.09	31	7.03		
	Total	229.03	34			
Necrosis surface of flap (cm2) Ratio	Between Groups	78.49	3	26.16	2.53	0.07
	Within Groups	319.70	31	10.31		
	Total	398.19	34			
	Between Groups	0.09	3	0.03	3.64	0.02
	Within Groups	0.25	31	0.008		
	Total	0.34	34			

## DISCUSSION

The most important ways to prevent flap ne-crosis are proper design, attention to width-length ratio, proper handling of tissue and he-mostasis, exact patient selection, cessation of smoking and treatment of co-morbidities. There are numerous clinical and experimental studies concerning other modalities or pharmacologic agents that have beneficial effects on flaps survival. The design and execution of these studies to test the drug effect on the flap survival is complex regarding the therapeutic agent used, the timing of the treatment, the route of application, the animal species employed, and the repeatability of the study.^3^

Two important mechanisms have been suggested to potentiate post-surgical flap damage, i.e. neutrophil infiltration and vascular thrombosis.^12^ Platelets play a central role in vessel thrombosis. It may be venous or arterial. Exposure of subendothelial matrix and release of prothrombin factors result in localized platelet adhesion and platelet activation and platelet-rich thrombus generation.^16^ Venous thrombosis seems to be a more important cause of flap necrosis than arterial occlusion.^11^ Prevention of platelet aggregation is one of the most important pharmacotherapy to prevent vessel’s thrombosis and subsequent flap necrosis.^16^

Heparin, aspirin and clopidogrel can prevent vessels thrombosis and enhance flap circulation and survival. Enoxaparin inhibits the conversion of prothrombin to thrombin, which reduces the thrombin-mediated conversion of fibrinogen to fibrin and prevents the formation of clots. Enoxaparin has a potent anticoagulant activity, but produces a lower propensity for bleeding when compared to unfractionated heparin.^19^

In addition to its anticoagulant properties, enoxaparin in low doses has anti-inflammatory effects, both in animal models and in human diseases. The beneficial effect of enoxaparin may be due to a combined anticoagulant and anti-inflammatory effects.^10^ Some authors assume that the mechanism behind this effect of enoxaparin is both anticoagulant and anti- inflammatory effects, but others propose that the anticoagulant property is the main factor, based on microcirculatory changes.^19^

The antiplatelet effect of aspirin is attributed primarily to inhibition of platelet cyclooxygenase-1, which is irreversible, resulting in reduction of the synthesis of thromboxan A2(TxA2) and consequent TxA2- induced platelet aggregation.^16^,^19^

Clopidogrel is a new antiplatelet medicine that was approved in 1997. At that time, its mechanism of action was not known. Great inter individual variability in effect was recognized very soon. Differences in oral absorption, variable metabolism, failure to clear the active metabolite, and differing ADP receptor reactivity may each participate in variability of the effect. Evidence supports variable oral absorption as a prominent factor.^14^,^15^ Clopidogrel is a prodrug that changes to its active metabolite after interaction with hepatic cytochrome enzyme P2C19 to inhibits platelet aggregation through irreversible blockade of P2Y12 (ADP) receptors on platelets.^14^,^15^,^19^-^21^

In one study that evaluates the effects of clopidogrel versus aspirin in patients at risk of ischemic events, clopidogrel therapy was more beneficial than aspirin specially in patients with peripheral arterial disease.^16^ There are many experimental and clinical studies that showed the beneficial effect of LMWH and aspirin on flap survival, although some others showed no effect. To our knowledge, there are only two experimental studies that evaluated the effect of clopidogrel on flap viability.^17^,^18^

Also there are a lot of studies that evaluated combined anticoagulation therapy. Some studies revealed that simultaneous inhibition of both pathways would provide a superior anti- thrombotic effect compared with single pathway inhibition.^15^,^22^ In one study, the combination of aspirin plus clopidogrel lead to an enhanced anti-thrombotic effects.^22^

Study of Khouri *et al. *suggests that heparin anticoagulation rather than aspirin is the key to maintaining micro-anastomotic patency.^23^

However, Savoie *et al. *studied thrombus composition used electron microscopy for thrombus composition analysis suggest that clinical use of two agents simultaneously, one inhibiting fibrin strand formation and the other inhibiting platelet adherence aggregation produce better results.^24^

However, anticoagulation is not without significant risk. The most important complications of platelet inhibiting agents are increased risk of bleeding especially in gastrointestinal tract. When we used combined therapy, the risk of complication increased. In two previous studies, clopidogrel produced a significant increase in survival rates of random flaps in rats, most probably through prevention of platelet aggregation. Vasodilatation, prevention of free O2 radicals and effect on ischemic reperfusion may be other mechanisms for the improvement noticed in the viability of flaps.^17^,^18^

Our results indicated a significant increase in flap survival in rats with clopidogrel administration. In our study, although low molecular weight heparin increased the flap survival area but the difference was not significant. When we combined both drugs, for some reasons, yet to be clarified, the survival area of flaps increased, but the difference was not significant. No clinical benefit was noted in combination with clopidogrel and low molecular weight heparin. Further study of these agents in experimental animals is justified.

This study showed that clopidogrel is an effective pharmaceutical agent that significantly increases viability of random skin flaps in rats, but low molecular weight heparin and the combination of the two drugs do not have any significant beneficial effects. This study had limitations that should be taken into account when interpreting the results. Experiments performed in rodent animals may not accurately predict the results in human beings. We are not sure about oral absorption of the drug in rats and did not measure serum concentration level of clopidogrel and its active metabolites. Also we did not collect information about adverse reactions especially increased bleeding tendency.

## CONFLICT OF INTEREST

The authors declare no conflict of interest.
